# Alarming and/or Alerting Device Effectiveness in Reducing Falls in Long-Term Care (LTC) Facilities? A Systematic Review

**DOI:** 10.3390/healthcare7010051

**Published:** 2019-03-25

**Authors:** Michael Mileski, Matthew Brooks, Joseph Baar Topinka, Guy Hamilton, Cleatus Land, Traci Mitchell, Brandy Mosley, Rebecca McClay

**Affiliations:** 1 School of Health Administration, Texas State University, San Marcos, TX 78666, USA; mb96@txstate.edu (M.B.); josephtopinka@txstate.edu (J.B.T.); ghamilton@edenhill.org (G.H.); shaneland5@gmail.com (C.L.); tracimitchell80@gmail.com (T.M.); butt3rfly@outlook.com (B.M.); 2 School of Science, Technology, Engineering, and Math American Public University System, Charles Town, WV 25414, USA; rebecca.mcclay@gmail.com

**Keywords:** falls, alarms, skilled nursing, quality improvement, safety

## Abstract

Perceptions against the use of alarming devices persist in long-term care environments as they are seen as annoying, costly, and a waste of time to the staff involved. Ascertaining whether these perceptions are true or false via the literature was a focus of this study. Proper information to educate staff and to work past these perceptions can be a positive effector for resident safety. Many facilitators for the use of alarming devices were found, as well as many barriers to their use as well. New technology is changing the perceptions regarding these types of devices as time passes. Education is a key component for staff, residents, and families. There are “traditional” issues with the use of alarms such as alarm fatigue by caregivers, high costs of implementation, and issues with proper implementation of alarms. Alarms are perceived as intrusive and the noise from them can be a potential cause of falls. However, alarming devices can be a key intervention in the safety of those residents who are prone to falls. This requires proper implementation and education for all parties involved, and proper oversight surrounding use of the devices.

## 1. Introduction

Falls within long-term care (LTC) facilities are a growing concern that cannot be ignored. On average, we see 1.6 falls per bed per year, with nearly half of those residents affected falling on multiple occasions [1,2,3,4,5]. Despite the countless prevention and intervention methods that have been deployed, falls remain the number one issue plaguing our LTC facilities [2,6]. This research contemplated the use of alarming and/or alerting devices that could aid in the reduction of falls. While many falls are located within the confines of the resident room, where they are out of the staff’s line of sight, alarming and/or alerting devices should aid staff in potentially preventing a fall [7].

### 1.1. Background

Alarming devices have long been a point of contention for residents, families, staff, and regulators of nursing facilities. The contention comes from beliefs on the use of such devices and whether they are effective means of allowing the staff to know that a resident who is utilizing one is in an unsafe situation [1,6,7,8,9]. Bed or chair alarms have been traditionally used to alert personnel when at risk patients are attempting to rise from a chair or their beds without assistance [7]. This study focused on looking at easing the contention and providing a basis of use for alarming devices in the nursing facility. Previous research in this area has been relatively scant, and often has shown little to no agreement for or against the use of alarming devices. Empirically speaking, many are against the use of alarming devices because they are loud, could possibly cause issues with behaviors in residents due to the sounds, and the perception that by the time they are going off, it is too late to intervene. There is also much empirical evidence to show benefits as well. This paper hopes to provide some specific evidence to show the effectiveness of alarms and how they can benefit residents of nursing facilities.

Various programs have been implemented and researched to help inform nursing staff of ways to identify high-risk residents, so that fall rates may be reduced. Largely, prevention measures that are applicable to the environment, when applied consistently, can aid in reducing resident falls [2,5,8,10]. Technology is a large area of focus today. The use of technology in the prevention of falls has met with much interest as technology continues to improve and we are beginning to see improved outcomes from its use [11,12,13,14]. Improved technology is reducing many of the “fear factors” of alarming/alerting devices of the past, such as false positives, decreased accuracy, or inappropriate reporting [11,14,15,16,17]. 

### 1.2. Significance

With the onslaught of the Baby Boomers, it has become more important than ever for facilities to become more proactive when it comes to resident fall intervention. It is estimated that by the year 2020 there will be in excess of 17.3 million falls per year, with an attached cost to healthcare of an astonishing $85 billion dollars [18]. By comparison, in the year 2000 falls costed an estimated $19 billion dollars in the United States [19]. It is easy to see how this is not only a safety issue, but also a financial one as well. Finding ways to mitigate the risk, on all fronts, must become an imperative.

Fall prevention and intervention is most effective when a holistic approach is deployed to identify those who are at a risk of falling and multifaceted intervention and prevention measures are put in place to reduce these chances [6,8,9]. To aid in this endeavor, technology must be brought into the fold as an intervention/prevention method. This study focused both on facilitators and barriers to these methods.

### 1.3. Objectives

This study focused on reviewing alarming and/or alerting devices and their potential effectiveness in reducing falls in LTC facilities. The long-term care industry adopted this technology (blindly, in some cases) hoping it would have the impact of improving resident care and reducing costs associated with falls. In an industry characterized by diminishing reimbursements, expensive technology with a relatively short life cycle must be able to prove that it can provide the intended results. This study was designed to review current literature related to the topic and was intended to provide a well-developed view of the efficacy of the technology as it relates specifically to reducing falls.

## 2. Materials and Methods 

**Figure 1:**
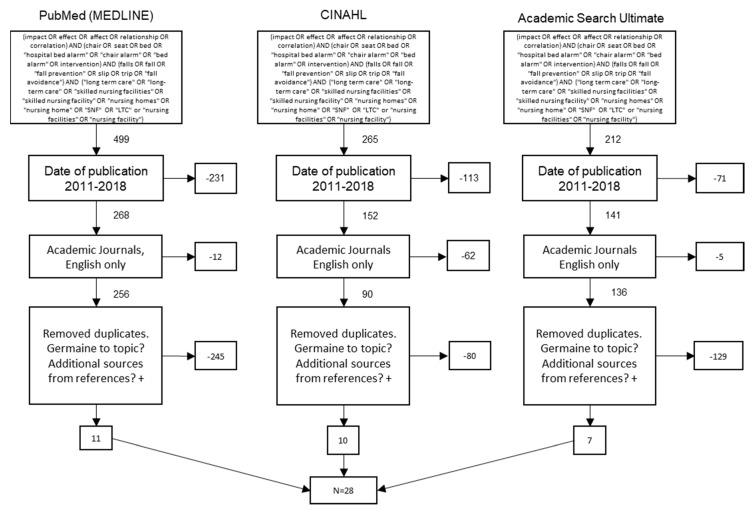
Preferred Reporting Items for Systematic Reviews flow diagram.

This study used a systematic review of peer-reviewed articles found in indexed databases. The Preferred Reporting Items for Systematic Reviews (PRISMA) guidelines were used to ensure consistent and precise reporting of results. The initial search was conducted using the Cumulative Index of Nursing and Allied Health Literature, Academic Search Complete, and PubMed. The Medical Subject Headings at the national Center for Biotechnology Information were used to initially identify keywords for queries. This information was then added to as our search progressed. This was done to avoid artificially narrowing the scope of our Boolean search of the databases. Our understanding and use of the most commonly used keywords led to the complex four-string Boolean search included in Figure 1. 

### 2.1. Inclusion Criteria

Authors individually reviewed the articles from the search, determined germane literature, and summarized themes. Inclusion criteria included English language and peer reviewed articles published in academic journals or by universities/colleges between 1 January 2011 and 31 December 2018. Articles had to explore the effects of bed or other alarms, fall prevention, and LTC style environments.

### 2.2. Exclusion Criteria

Articles were incorporated into this study only if deemed germane by all authors. Trade industry reports and poster presentations without clear, scientific format, and a peer review process were excluded. Articles which were not specific to LTC environments were excluded. Similar examination outcomes demonstrated that the authors had a parallel understanding of the research subject and scope. Bias was not considered when reviewing the research involved in this study. The final sample of articles after meeting exclusion criteria was then analyzed for further consensus among all authors for final inclusion. When analyzed, this sample yielded a kappa statistic (*k* = 1), showing strong reliability between researchers.

### 2.3. Study Selection 

The article selection process is outlined in the PRISMA diagram in Figure 1. The initial search resulted in 976 results. The scope was then narrowed via a reduction of the publication time frame to between 2011 through 2018, leaving 561 articles. The scope was then further reduced to only academic journals and articles in the English language, leaving 482 articles. Authors then reviewed abstracts and removed duplicates and non-germane articles from the search, eventually leaving 28 articles for use in this review.

### 2.4. Data Analysis

Narrative summaries related to factors that influenced the use of alarms in nursing facilities were extracted from each article. These, in turn, were grouped into larger recurring themes that were either key determinants or impediments to the use of alarming devices. The themes chosen were done so by consensus of the authors. Those themes chosen were agreed upon to be ones that provided overarching summary to the facilitators and barriers extracted. These themes were then divided into two affinity matrix tables for facilitators and barriers. Each table documents the themes, their citation occurrence, their frequency sum, and frequency percentage.

## 3. Results

Overall, 118 factors of facilitation (57, or 48%) or barriers (61, or 52%) were observed. We identified ten facilitation themes and eight barrier themes. The detailed list of facilitators and barriers to adoption from each study are shown in Table S1.

### 3.1. Facilitators

Overall there were slightly fewer facilitators than barriers noted (57:61). Bed and chair alarm technology is constantly changing and improving [18]. Alarms are becoming more efficient and even wearable [11,12,13,14,15,16,20,21]. Improvements in type and the implementation of new alarm systems require sufficient staff training [19]. The appropriate use of alarms on the appropriate population of residents in the long-term care facility must be considered if alarms are to be fully leveraged [7,11,12,16,18,22,23,24,25,26,27,28,29,30]. Alarm maintenance is critical, and alarms must be one part of a more comprehensive plan of care to be most effective in preventing falls [18,24,25]. Alarms have proven effective with the elderly population at risk of falling [7,11,12,16,17,18,23,24,25,28,29,30]. Alarms can create an increased sense of security in residents who suffer from cognitive impairments or who are at greater risk of falling [26,27,31].

Alarms are most impactful in reducing falls for residents with cognitive impairments [7,11,12,15,17,18,19,21,22,23,24,28,32,33,34,35]. Alarms also reduce the burden of care placed upon staff [15,17]. Staff of nursing facilities especially have a favorable view of bed and chair alarms [15,17]. Staff report that alarms provide an opportunity to provide support when it is needed [18,22,24,28]. Additionally, alarms seem to increase the likelihood of resident and staff interaction, which is directly related to quality of care [17,22,23,28,30]. Improvements in alarm technology are leading to increased device accuracy and increased resident quality of life [11,15,34]. With proper training and implementation, bed and chair alarms have been shown to improve resident quality of life and nurse efficiency [22,24,25,26,27,31].

Table 1 illustrates and ranks the orders of themes from analysis of the facilitators of the use of bed and chair alarms and other alerting devices to reduce the number of falls in long term care facilities based on the frequency of their occurrence in the literature.

The theme most often mentioned was ’proper implementation of alarms improves care’, identified in 17 out of 57 total occurrences (29.8%) [7,11,12,16,17,18,22,23,24,25,28,29]. Alarms were never meant to be a standalone intervention to prevent falls but rather to be one part of a comprehensive plan to assist with the monitoring of residents with increased fall risk [11,18,22,23,24,25,30]. Fall risk, which may be evaluated objectively by rating tools, would then direct caregivers to those residents who may benefit most from some type of monitoring system [7,12,17,22,24,25,30]. A direct and effective notification system should be in place to support the alarm system’s ability to notify a specific caregiver [11,12,17,23,28,29,30,33]. All staff should be thoroughly trained on the proper implementation of the device [18,22,30]. This should include all portions from connection to resident placement. Devices should be installed properly and monitored effectively by trained staff [7,11,18,30]. Under these circumstances, alarms are a much more effective strategy to prevent falls.

‘Technology improvements have increased alarm efficacy’ was a theme also mentioned often, in 16 out of 57 total occurrences (28.1%) [11,12,13,14,15,16,17,20,21]. Many aspects of alarms that were undesirable are beginning to evolve with improvements in alarm technology [12,13,14,16,17,21]. Alarm devices are becoming more accurate due to improvements in infrared and wearable technology, which allows engineers to reduce the incidence of false positives [11,13,14,15,16,17,20]. Smaller and more comfortable alarm devices also have the capability of sending a direct alert to a specific caregiver [11,12,16]. This reduces staff burden and increases the efficacy of the alarm system, which will broaden its application [14,16,20,21]. Improvements in alarm technology are also providing for more inexpensive alarm options in the market [17].

The theme ‘proper implementation of alarms reduces fall risk’ was noted in 10 out of 57 occurrences (17.5%) [15,17,19,28,32,34,35]. As more is learned about alarms in general, an increasing amount of data has been gathered concerning the most effective implementation of alarm systems and how important it is for alarm-use success. Several studies mentioned how the proper use of alarms was an effective intervention for those with confusion, agitation, or dementia [15,17,28,32,34,35]. Equally, the effective manipulation of the resident environment to ensure that walkways, bed areas, and use areas are clear of hazards helped to increase alarm efficacy [19,32]. Proper identification of residents who would benefit the most from the use of properly implemented alarms was also a factor [17,32,34,35]. Alarms should be part of a comprehensive and well-designed plan of interventions for each resident.

‘Improved resident quality of life’ was mentioned in 6 out of 57 occurrences (10.5%) [26,27,31]. Alarms and other monitoring systems have been shown to provide added peace of mind to elderly residents who have a fear of falling [26,27,31]. When surveyed, residents noted an improved sense of well-being knowing that the alarm was there to assist them [26,27,31]. Alarms were noted to correlate to increased numbers of resident and caregiver interactions, which leads to higher quality resident care and improved quality of life [26,31]. Caregivers reported being able to intervene more effectively and assist residents who were at higher risk of falling due to the alarm system notifications to them [26,27].

‘Resident education about falls is important’ was another theme and was present in 2 out of 57 occurrences (3.5%) [18,36]. Both studies showed a positive impact on the acceptance of alarms and the likelihood of residents to wear the alarm monitors if they understood their personal fall risk and the negative impact on their health. Higher awareness by residents through alarm use can lead to a greater safety awareness [18]. Residents being able to become self-aware with the use of alarms provides necessary feedback to them to assist in their own fall prevention [36].

‘Proper implementation of alarms improves alarm efficacy’ was another theme present, which occurred 2 out of 57 times (3.5%) [33]. Researchers noted that for alarms to work properly they must be properly installed, maintained, and understood by staff [33]. This can be implemented via the use of bed mats, bed sensors, or lightweight technologies that can predict bed exits, which can alert the staff to act early [33].

‘Improved care for residents with cognitive impairments with alarms’ was mentioned in 1 out of 57 occurrences (1.8%) [27]. Cognitive impairments included residents with certain types of dementia and those with general cognitive issues resulting from various etiologies. The improvement of supervision in this population was mentioned due to their lack of communicative ability and in some cases personal awareness. The fact that these residents were in a new or different environment equated to an increased fall risk for them [27]. Understanding this allows the staff to properly implement care plans to decrease the number of falls.

Other themes discovered were ‘staff in favor of alarms’ [15], ‘staff interaction with residents reduces fall risk’ [21] and ’staff training in alarm use’ [19]. Each of these themes occurred once (1.8%). Staff noted a decreased burden and higher level of interaction with their most at-risk residents [15,19,21]. Additionally, proper training in how to apply alarms and how to assess fall risk was also stressed as being important to implementation [19,21].

### 3.2. Barriers

Barriers to alarming and/or alerting devices reducing falls in long-term care are illustrated in Table 2. Overall, there were slightly more barriers to alarm effectiveness in preventing resident falls than there were facilitators (61:57). Of the identified barrier themes, there was an overwhelming number related to alarms being ineffective. Whether this ineffective nature came from the alarm itself as a standalone intervention, fatigue caused to caregivers due to alarm use, or staff becoming habituated to their sounds to the point where they did not recognize/pay attention to them anymore, these are all very significant areas when it comes to alarm use. Identifying these areas as problematic is just as important as identifying the facilitators previously mentioned. Only the consideration of the positive and negative features of the use of alarms will assist staff in using them as an effective fall-prevention strategy. 

Table 2 illustrates and ranks the orders of themes from analysis of the barriers of the use of bed and chair alarms and other alerting devices to reduce the number of falls in long-term care facilities based on the frequency of their occurrence in the literature.

The theme ‘ineffective as standalone intervention’ [12,13,15,16,19,21,22,23,24,25,27,28,30,31,32,36] was mentioned in 23 out of 61 occurrences (37.7%). Many staff raise concerns over alarms and having no way of monitoring if they are correctly working throughout a shift [22]. There are also concerns over the proper implementation of alarms by staff who set them up initially, and those who manipulate them later during provision of care [23]. The perception (and evidence) that alarms for decreasing falls simply do not work is pervasive in the literature [19,22,23,28]. It is also clear that alarms by themselves do not seem to be effective as a fall prevention measure [7,19,21,24,25,30]. Alarms work best as part of a more comprehensive program that has other interventions in place in addition to the alarm [19,21,30]. It is clear that this is an area that requires further research [30]. When considering overall evidence for or against alarms, there are inconsistencies across studies as to the effectiveness [13,27,28]. Concern also exists that there is no association between alarm use and fall rates, which shows their ineffective nature [27,28]. Alarms also do not take into consideration other issues which may exist which can contribute to falls, such as inadequate depth perception or transferring deficits [16]. There are no alarms which can assist with these areas.

The theme ‘alarm fatigue by caregiver’ [14,16,17,21,22,23,26,33,34] was mentioned in 12 out of 61 occurrences (19.7%). Nurses raised valid concerns over the use of alarms and the placement of residents on their halls. There is a practical perception that alarms become less effective when there are too many at use in a facility at one time, or if the residents using them are isolated [22]. The sounding of alarms can become very tiring for staff members who are responding when it happens frequently [23], or if the alarms are falsely sounding [14,23,26]. False alarms not only contribute to staff fatigue needlessly, but they also force the staff to question alarm usefulness [14,16]. The types of alarms used contribute in this area as well, as “traditional” types of bed alarms are apt to give false signals, causing staff to respond when there is no valid reason to do so [16,21,33].

The ‘theme difficult to implement properly’ [7,12,14,15,16,18,28,37] was mentioned in 8 out of 61 occurrences (13.1%). Issues surrounding equipment malfunctions were mentioned repeatedly in the literature [14,15,16,18]. Some of these malfunctions come directly from the lack of proper implementation and use of the alarm by the staff, whether it be from alarm issues or lack of training [7,15,18,37]. The issue of resident comfort versus resident safety is also of concern in this area, as bed positioning can contribute to issues with alarms properly working [15,16,18,20,37]. Residents can also cause issues with alarms if they themselves are in an incorrect position for them to work properly or their movements cause leads to fail or become dislodged, resulting in alarm failure [14,18,37]. Concern also exists that alarms do not activate quickly enough, as alarms have been reported to go off after the resident has left their chair or has already fallen [16].

’Expensive to implement’ [16,18,28,33,35] was mentioned in 5 out of 61 occurrences (8.2%). There is a great cost associated with newer, high-tech systems in both home care settings and long-term care settings. Using existing facility infrastructure, it is often not feasible to implement alarms, which causes a significant increase to costs in use and installation [16,18,33,35]. A lack of existing infrastructure often leads to facilities having to use very expensive RFID technologies to implement an alarm solution [35]. Literature was also directed around cost effectiveness being an issue in acute care settings [28], regarding inventory and maintenance of the systems [16], and in continuing development of the systems keeping them effective and on the cutting edge of technology [33,35].

‘Increased staff burden’ [15,18,22,29] as a barrier was mentioned in 4 out of 61 occurrences (6.6%). Learning new equipment and systems costs time and money [15,22,29]. Resources available to long-term care providers are constrained, which can cause issues with large-scale implementation when it comes to an already overburdened staff [22,29]. Nurses tend to focus on tasks perceived as more important, as opposed to expending the time to respond to alarms [15,29]. Dealing with common equipment malfunctions also causes increased nursing workload and causes their time to be capitalized by alarm needs as opposed to other important resident needs [18]. 

The themes ‘alarm noise annoyance’ [11,17,23], ‘noise of alarms startles residents’ [11,23], and ‘resident reluctance’ [26] were each mentioned 3 out of 61 times (4.9% each). Alarm noise annoyance creates a not-so-pleasant environment in which to live when alarms are going off at all hours of the day and night, causing issues for not only those requiring alarms, but also other residents as well [17,23]. When these alarms were integrated into the nurse call system, this caused increased disturbances in the facility [17]. Another concern was alarms sounding when normal activities of daily living were being attempted, despite residents not attempting activities which are designed to cause the alarms to sound [17]. Alarms were noted to cause annoyance among residents, visitors, and caregivers [26]. This perception caused residents to not want to use the alarms as they perceived themselves as a burden on others [26]. Noise of alarms has also been noted to startle residents and can have an adverse effect on resident safety [11,23,26]. It has been argued that the sound of the alarm can startle or scare a resident and cause, attribute to, or lead to a fall [23]. Residents may also be reluctant to wear and/or use the fall-prevention device as they can feel embarrassed using the alarm or do not want to lose their independence [26].

## 4. Discussion

The objective of this study was to review the efficacy of alarms and alarming devices in reducing falls in long-term care facilities. Overarching political debate regarding budgetary spending and the nation’s deficit are driving policy makers to pursue methods to reduce spending wherever possible. Medicare and Medicaid spending make up a bulk of budgetary expenditures in healthcare, especially in long-term care, where many of these alarms are commonly used. As the population of the United States stands on the precipice of one of the largest elderly populations in history, spending on healthcare for this population has been the subject of considerable debate both politically and academically.

We understand that falls are a leading cause of added healthcare expenditures, especially in elderly populations [2]. “Falls are the leading cause of fatal and nonfatal injuries among adults aged ≥65 years (older adults). During 2014, approximately 27,000 older adults died because of falls; 2.8 million were treated in emergency departments for fall-related injuries, and approximately 800,000 of these residents were subsequently hospitalized” [1]. Not only is this a concern by caregivers, but it has also become a public health concern as well.

Seemingly, many of the barrier themes presented here could be overcome by the facilitators presented if the appropriate amount of resources were available to develop alarming technologies. We can only draw conclusions based on conjecture as to why alarming technologies did not work in specific facilities, or with particular types of caregivers. We can only make assumptions based on the negative literature against their use. We can make the same leap with all the positive literature as well. Alarms and alarming devices may hold a key to helping caregivers reduce falls. This being said, alarms in their current form are simply not advanced enough to act as a standalone intervention to prevent falls and they likely never will be. However, when applied in a responsible manner with residents who are at most risk for falls and with specific communication protocols, alarming devices and alarms could be one of the most promising technological investments we could make to support our elderly.

## 5. Conclusions

One overarching conclusion is easily made from this review, and that is that bed alarms alone are relatively useless as an intervention. For alarms to be effective, they must be part of a much more comprehensive care plan put into place for residents who are at risk for falling. In fact, there is no single thing that can be done which reduces fall rates. Fall rates which are reduced in any facility are reduced by a multi-pronged approach to resident safety and by a clear focus by staff on keeping residents safe.

Staff perceptions are mixed when it comes to the use of alarming devices. Staff conclude that they work, and that they also do not. They can be burdensome and cause issues for staff in general who are caring for residents who use them. Studies in this area, however, are unclear as to the staffing situations in these facilities or the resident acuity. In fact, this is an area that has been left untouched in the literature in the assessment of alarming devices. Perceptual studies are aplenty; however, there is little information on the climate of the facilities where those perceptions are coming from, making their negative findings questionable. This is an area where study needs to occur for certain to better understand the negativity surrounding alarm use.

Overall, it seems that alarm use can assist in the care and safety of nursing facility residents (Table 3). When used as one piece of a comprehensive care plan, they can be quite effective devices in ensuring resident safety. While further studies are necessary, we currently have an effective intervention which can be used to assist in the care of those living in nursing facilities.

## Figures and Tables

**Table 1:** Themes associated with alarms and fall reduction.

Facilitators	Occurrences	Sum	%
Proper implementation of alarms improves care	[7,11,12,18,22,23,24,25] *, [16,17,28,29]	17	29.80%
Technology improvements have increased alarm efficacy	[11,14,17] *, [12,13,15,16,20,21]	16	28.10%
Proper implementation of alarms reduces fall risk	[15,32] *, [17,19,28,34,35]	10	17.50%
Improved resident quality of life	[17,31], [26] *	6	10.50%
Resident education about falls is important	[18,36]	2	3.50%
Proper implementation of alarms improves alarm efficacy	[33] *	2	3.50%
Improved care for residents with cognitive impairment with alarms	[27]	1	1.80%
Staff in favor of alarms	[15]	1	1.80%
Staff interaction with residents reduces fall risk	[21]	1	1.80%
Staff training in alarm use	[19]	1	1.80%
Total		57	100%

* multiple occurrences mentioned in one article.

**Table 2:** Themes associated with alarms and fall reduction.

Barriers	Occurrences	Sum	%
Ineffective as standalone intervention	[16,21,22,23,24,28] *, [12,13,15,19,25,27,30,31,32,36]	23	37.70%
Alarm fatigue by caregiver	[17,22,23,26,34], [14,16,21,33] *	12	19.70%
Difficult to implement properly	[7,12,14,15,16,18,28,37]	8	13.10%
Expensive to implement	[16,18,28,33,35]	5	8.20%
Increased staff burden	[15,18,22,29]	4	6.60%
Alarm noise annoyance	[11,17,23]	3	4.90%
Noise of alarms startles residents	[23] *, [11]	3	4.90%
Resident reluctance	[26] *	3	4.90%
Total		61	100%

* multiple occurrences mentioned in one article.

**Table 3:** Summary of themes associated with alarms and fall reduction.

Summary of Facilitators	Summary of Barriers
Nurses perceived bed/chair alarms as a useful way to prevent resident falls.	Alarms must be consistently monitored and kept in working order.
Alarm systems which send signals directly to caregivers for immediate action are more effective in use.	Concerns exist when alarms are used on residents who are isolated from others, or if there are too many alarms in use in one area.
Bed alarms as part of a comprehensive fall prevention plan reduce falls.	Alarms alone do not reduce fall rates.
The use of infrared beam detectors along with alarms can promote the timely activation of the alarm.	Alarms can startle residents, causing negative effects from this reaction, and could potentially cause a fall. These loud sounds may cause a flight response in residents.
Tailoring the alarm type to resident characteristics can improve alarm performance and ultimately reduce the risk of falls and related injuries.	Alarms can be viewed as loud and burdensome to other residents because of their environmental impact.
Alarms using dual pressure sensors plus infrared beam detectors were more accurate than only pressure sensitive alarms in identifying bed-exiting.	Equipment malfunctions associated with medical technology can increase workload for nurses.
Fall prevention initiatives and activities must be in the forefront of all activities in the facility for the best effectiveness.	Bed and chair alarms must be implemented correctly to reduce fall rates. Otherwise, they could be contributors.
Resident education about fall prevention and safety awareness can also be an effective method at reducing falls.	False alarms can be burdensome on staff and residents and can contribute to "alarm fatigue" by staff members.
Focus on resident self-assessment and feedback regarding fall risk can assist in reducing falls.	Devices became a “nuisance” to residents because of their functionality and application.
Use of monitoring system can have a positive impact on resident quality of life.	
Residents with a fall risk can have a greater sense of security when using alarming systems.	Residents may be reluctant to wear alarms because they do not want to inconvenience others or lose their independence.
Residents who wore their fall detector felt more confident and independent and reported that devices improved their safety and decreased their fear of falling.	No consistent evidence that current sensor technology reduces fall rates
Bed-exit alarms used with confused and agitated residents may have helped reduce falls.	Those who consider alarming-device restraints perceive these devices to contribute to a lower quality of life for residents and a potential source of additional injuries.
Bed height alerting systems can also have a positive impact upon reducing resident falls.	Residents are often able to disconnect the alarms on their own, rendering them useless.
Interventions such as a falls risk flag in the records/on beds, additional supervision when the resident is mobilizing or in the bathroom, keeping areas clear of hazards, and use of chair/bed alarms can reduce falls.	Cost effectiveness of alarms and alarming systems is a concern.
Reducing the number of unassisted transfers through a modest improvement in response time to alarms can positively affect fall numbers.	Alarm signals may occur after residents had already fallen because they fell immediately on exiting the bed or chair.
Coworkers aware of and assisting with alarm devices decreased the noncompliance rates resident alarms from 50 to 30 percent.	
Reduce false positives by using alarm systems that allow for adjusting sensitivities.	

## Supplementary Materials

The following are available online at https://www.mdpi.com/2227-9032/7/1/51/s1, Table S1: Literature review matrix.
